# Value of dual Doppler echocardiography for prediction of atrial fibrillation recurrence after radiofrequency catheter ablation

**DOI:** 10.1186/s12872-019-1233-x

**Published:** 2019-11-15

**Authors:** Fengjiao Chen, Qinliang Sun, Hairu Li, Shaohui Qu, Weidong Yu, Shuangquan Jiang, Jiawei Tian

**Affiliations:** Department of Ultrasound, The Second Affiliated Hospital of Harbin Medical University, Baojian Rd. 148, Harbin, 150086 China

**Keywords:** Atrial fibrillation, Radiofrequency catheter ablation, Dual Doppler echocardiography, Recurrence

## Abstract

**Background:**

Increasing evidence has been presented which suggests that left ventricular (LV) diastolic dysfunction may play an important role in the development of atrial fibrillation (AF). However, the potential for LV diastolic dysfunction to serve as a predictor of AF recurrence after radiofrequency catheter ablation remains unresolved.

**Methods:**

Dual Doppler and M-PW mode echocardiography were performed in 67 patients with AF before ablation and 47 patients with sinus rhythm. The parameters measured within identical cardiac cycles included, the time interval between the onset of early transmitral flow peak velocity (E) and that of early diastolic mitral annular velocity (e’) (TE-e’), the ratio of E to color M-mode Doppler flow propagation velocity (Vp)(E/Vp), the Tei index, the ratio of E and mitral annular septal (S) peak velocity in early diastolic E/e’(S) and the ratio of E and mitral annular lateral (L) peak velocity E/e’(L). A follow-up examination was performed 1 year after ablation and patients were divided into two groups based on the presence or absence of AF recurrence. Risk estimations for AF recurrence were performed using univariate and multivariate logistic regression.

**Results:**

TE-e’, E/Vp, the Tei index, E/e’(S) and E/e’(L) were all increased in AF patients as compared with the control group (*p* <  0.05). At the one-year follow-up examination, a recurrence of AF was observed in 21/67 (31.34%) patients. TE-e’ and the Tei index within the recurrence group were significantly increased as compared to the group without recurrence (*p* <  0.001). Results from multivariate analysis revealed that TE-e’ can provide an independent predictor for AF recurrence (*p* = 0.001).

**Conclusions:**

Dual Doppler echocardiography can provide an effective and accurate technique for evaluating LV diastolic function within AF patients. The TE-e’ obtained within identical cardiac cycles can serve as an independent predictor for the recurrence of AF as determined at 1 year after ablation.

## Background

Atrial fibrillation (AF) is one of the common cardiovascular diseases. Even in patients with good anticoagulation, AF can cause severe stroke, increased mortality, heart failure, and left ventricular (LV) diastolic dysfunction [1]. Recently, radiofrequency catheter ablation (RFCA) has become a common treatment for patients with symptomatic, drug-resistant AF. AF recurrence after the RFCA is defined as atrial fibrillation, atrial flutter or atrial tachycardia for 30 s or more after a 3-month blanking period without antiarrhythmic drugs, with recurrence rates being 30–50% [2, 3]. It has been demonstrated that LV diastolic dysfunction is related with recurrence of AF after RFCA [4, 5]. One approach commonly used to evaluate LV diastolic function in AF patients is echocardiography [6–9]. However, the accuracy of assessing LV diastolic function with parameters from different cardiac cycles as measured by conventional echocardiography is limited. Dual Doppler echocardiography represents a relatively new technique which has the capacity to not only provide information on discrete sites of blood flow and myocardial tissue movement within identical sections of the heart, but also to simultaneously acquire information on ultrasonic parameters within the same cardiac cycle. As a result, this technique provides a more accurate means for evaluating LV diastolic function, especially in patients with arrhythmias [10].

From the description above, it is clear that an important relationship exists between LV diastolic dysfunction and AF and that dual Doppler echocardiography can serve as an effective technique for assessing LV diastolic function. Therefore, the goals of this study were to use dual Doppler echocardiography as a means to: 1) determine the extent of LV diastolic dysfunction in patients with AF and 2) assess the predictive value of LV diastolic function for the recurrence of AF as determined at 1 year after RFCA.

## Methods

### Patients

From October 2016 to October 2017, a total of 67 AF patients who were about to undergo RFCA in the Second Affiliated Hospital of Harbin Medical University participated in our study, they were all with preserved left ventricular systolic function (left ventricular ejection fraction, LVEF > 50%). Exclusion criteria included intra-cardiac thrombi, structural heart disease, hyperthyroidism or other autoimmune diseases, rheumatic heart disease and LVEF < 50%. In addition, a total of 47 patients with sinus rhythm and LVEF > 50% were selected as control group, who came to hospital just because of primary hypertension, chronic coronary heart disease, or physical examination. The study complied with the Declaration of Helsinki and was approved by the Ethics Committee of Harbin Medical University. All patients signed a written informed consent form prior to participating in the study.

### Study protocol

**Fig. 1:**
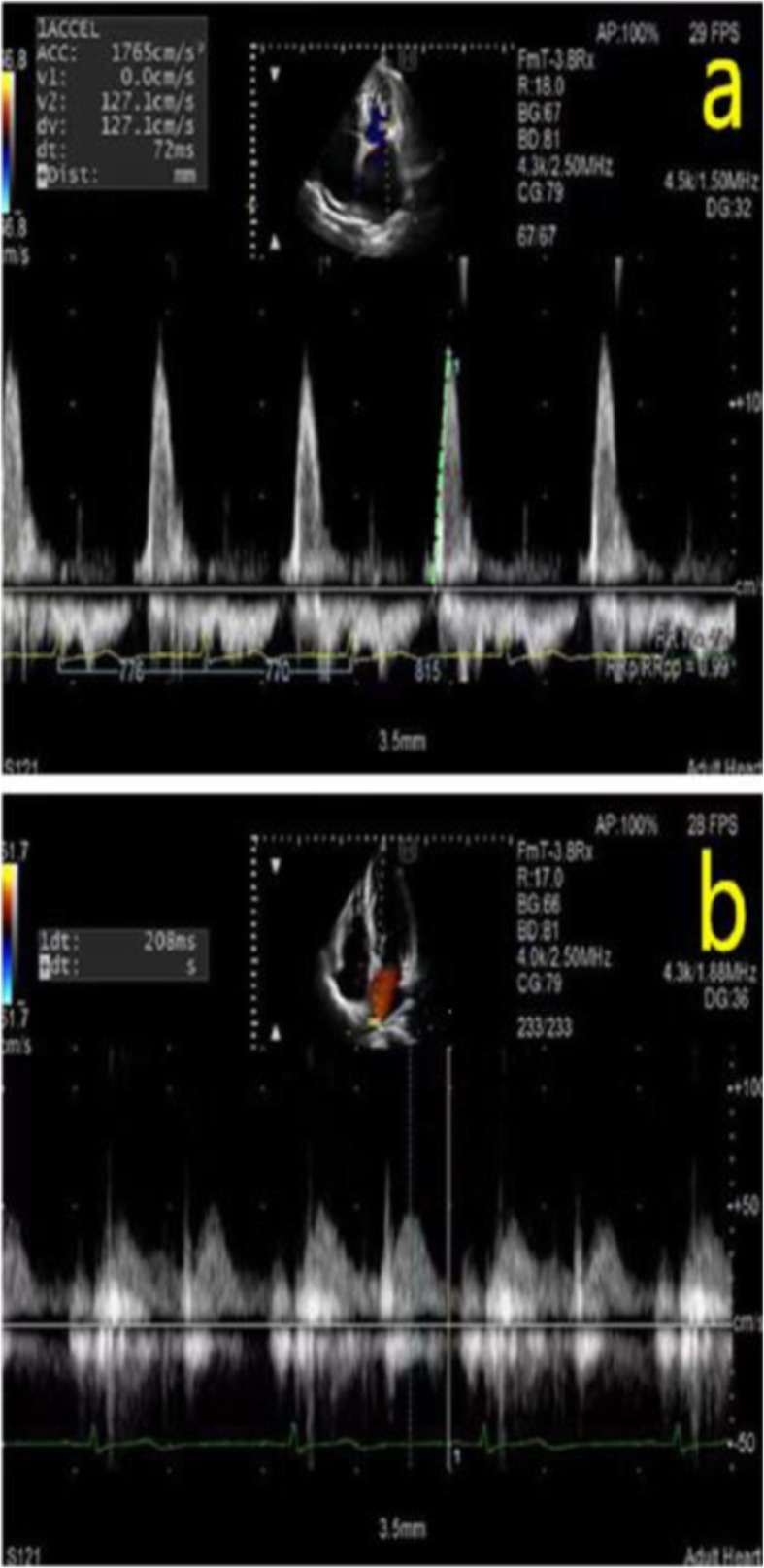
**a** Transmitral velocity was tracked and peak acceleration of the early transmitral flow peak velocity was measured. **b**. The flow of the right pulmonary vein was confirmed with the help of color doppler ultrasound in the apical four-chamber view and we measured deceleration time of pulmonary venous diastolic velocity

Baseline clinical information was collected and conventional echocardiography, M-PW mode echocardiography and dual Doppler echocardiography were performed on all subjects. Plasma NT-proBNP level was measured in the AF group. Both AF patients and the control group were subjected to transthoracic echocardiography using Aloka ProSound F75, Hitachi, Japan with a combined 1^_^5-MHz Doppler transducer. All patients underwent precordial 2-dimensional, M-mode and Doppler echocardiography while in the left lateral position and all images were stored on a hard disk for subsequent playback and analysis. Left atrial diameter (LAD), LV end-diastolic and end-systolic dimensions were measured from the M-mode echocardiogram. LVEF was calculated by the Simpson method using 2-dimensional images. LA maximum volume was obtained by manually tracing the LA endocardium on the apical four-chamber and the apical two-chamber views by Simpson method before MV opening. LA maximum volume divided by body surface area, we obtained LA maximum volume index. We also recorded the early transmitral flow peak velocity (E: cm/s) and the tissue Doppler-derived mitral annular peak velocity in early diastolic (e’:cm/s), both at the lateral annulus(L) and the interventricular septal annulus(S). Transmitral velocity was tracked and peak acceleration (PkAcc) of the E velocity was measured on the machine (Fig. 1a). The flow of the right pulmonary vein was confirmed with the help of color doppler ultrasound, and the sample volume was placed 0.5 to 1 cm into the vein to record the velocity of pulmonary vein flow in the apical four-chamber view and we measured deceleration time of pulmonary venous diastolic velocity (PV-DT) (Fig. 1b).

**Fig. 2:**
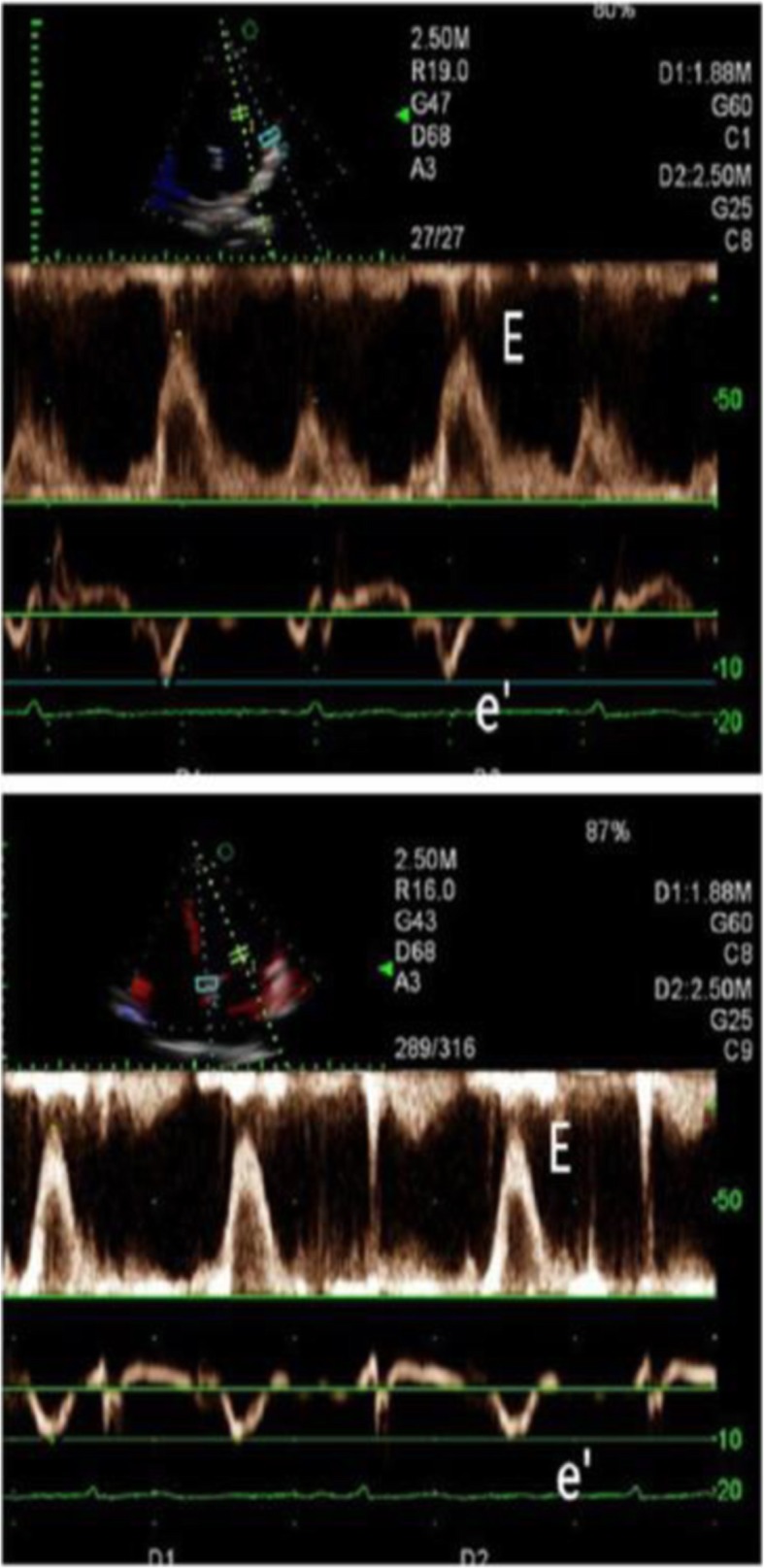
Measurement of transmitral inflow and the mitral annular velocity using a dual Doppler system. A total of 2 pulsed sample volumes were positioned between the tips of the mitral leaflets and the base of the left ventricular lateral wall or interventricular septum, and E and e’ were measured

**Fig. 3:**
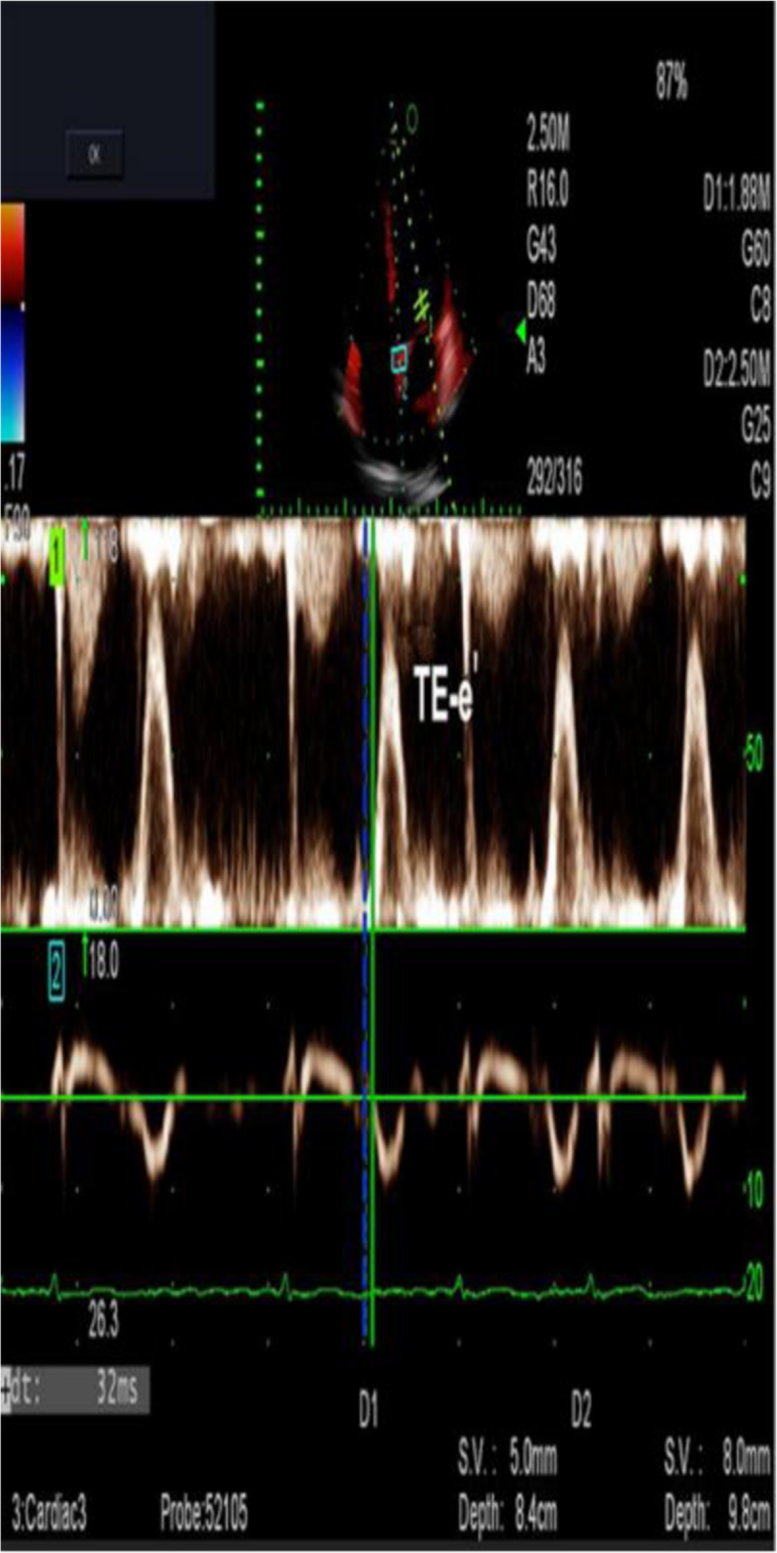
Measurement of TE-e’ using a dual Doppler system. From simultaneous recordings of transmitral inflow and mitral annular velocity, the time interval was measured between the onset of E and of e’(TE-e’) for 10 consecutive beats

**Fig. 4:**
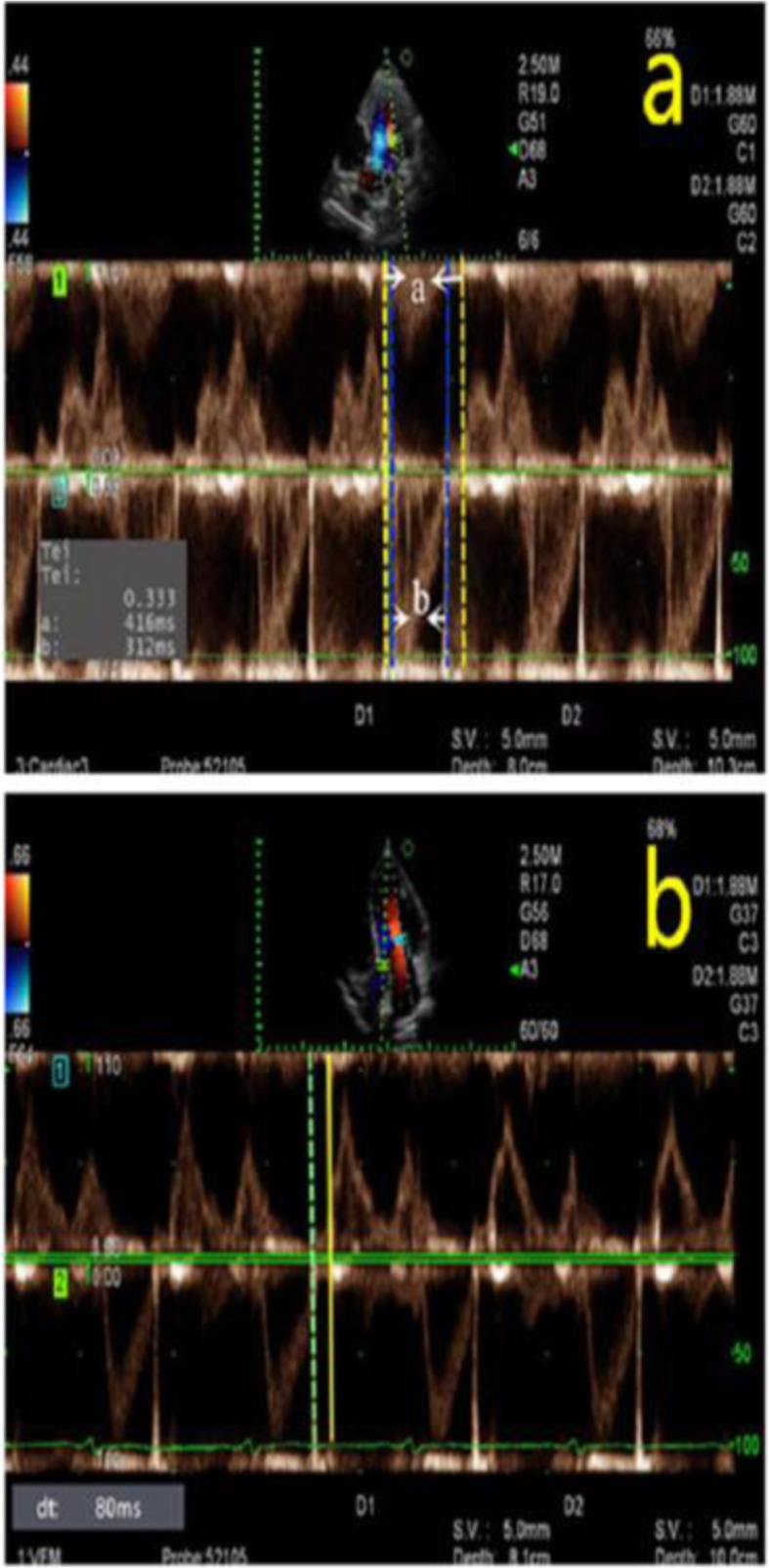
**a**. Tei index is defined by the equation (a-b)/b, where “a” represents the interval between cessation and onset of mitral inflow, and “b” represents the ejection time of the left ventricular outflow. **b**. The isovolumic relaxation time was measured by dual Doppler echocardiography, with the green imaginary line representing the end of aortic flow and the yellow full line representing the beginning of mitral flow

**Fig. 5:**
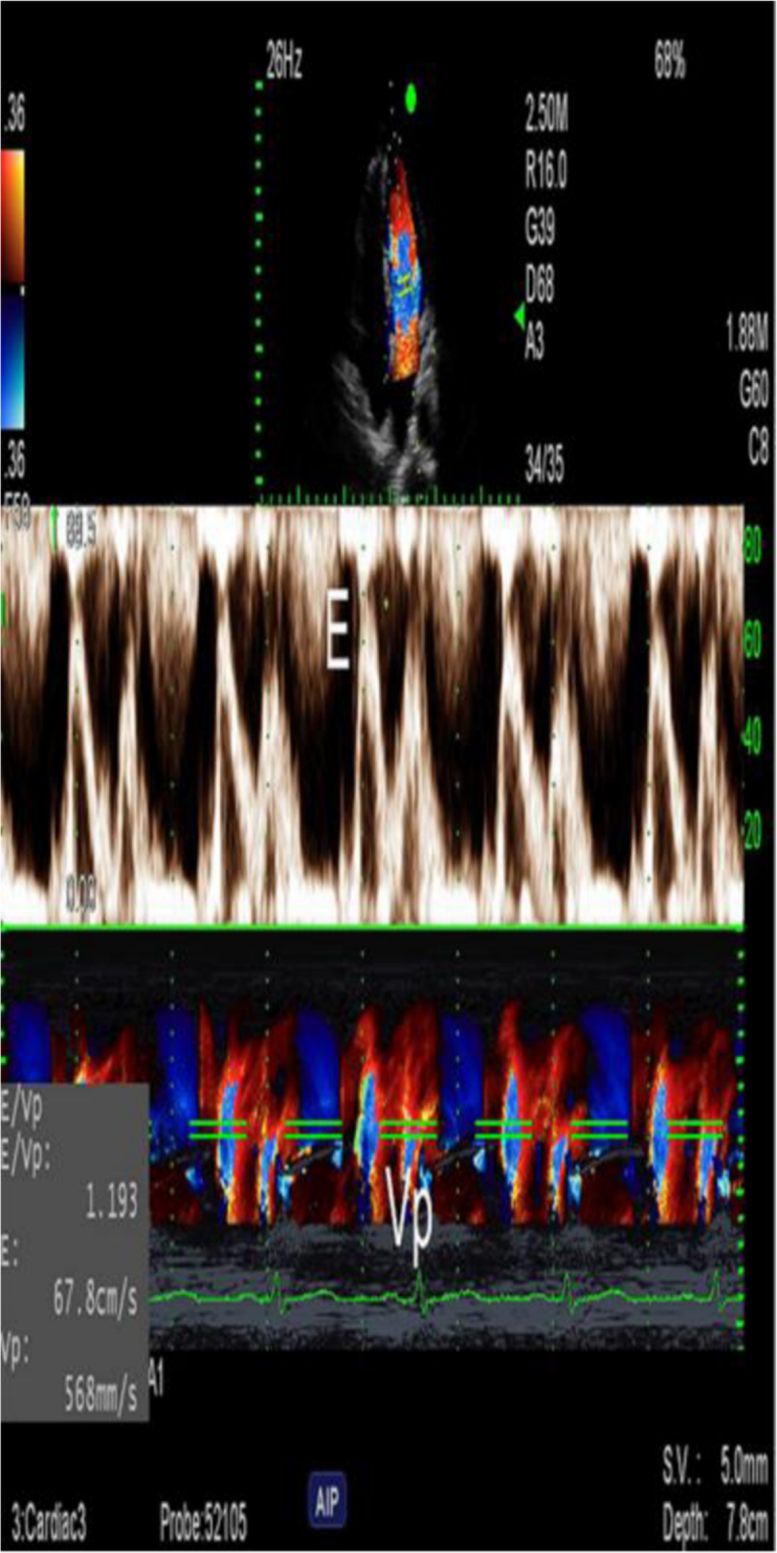
Early transmitral flow peak velocity(E) and Vp were measured in the same cardiac cycle using M-PW mode echocardiography. Vp was measured as the slope of the first aliasing velocity during early filling, from the mitral valve plane to 4 cm distally into the left ventricular cavity. E/Vp is the ratio of E to Vp

Images from AF patients and the control group were collected with use of dual Doppler and M-PW mode echocardiography at identical cardiac cycles in the apical four-chamber view and five-chamber view. Simultaneous recordings of E and e’ were performed using the dual Doppler imaging method (Fig. 2). As shown in Fig. 2, two doppler sampling lines were placed at the tips of the mitral leaflets and mitral annulus, first at the lateral and then at the septal corners of mitral annulus, we obtained E/e’(L) and E/e’(S) in identical cardiac cycles [10]. The time interval between E and septal e’ (TE-e’) was then measured (Fig. 3). The Tei index was measured in the apical five-chamber view, with one sampling line placed between the mitral valve leaflets and the other placed at the aortic valve. The Tei index was then calculated by subtracting the LV ejection time (b) from the time interval between cessation and onset of the mitral inflow (a) divided by the LV ejection time (a-b/b) (Fig. 4a) [11]. Isovolumic relaxation time (IVRT) was measured from the end of aortic flow to the beginning of mitral flow (Fig. 4b). Placed the M-mode cursor with the pulsed sample volume in the center of the mitral valve, avoiding the surrounding area and in the same direction as the flow stream. The pulse doppler sampling volume was placed at the tips of the mitral valve to obtain E velocity. The slope of the first aliasing velocity during early filling was measured as Vp, extending 4 cm from the mitral valve into the left ventricle (Fig. 5) [12, 13]. E/e’, TE–e’, the Tei index and E/Vp were measured for 10 consecutive beats and the values were averaged.

All AF patients were required to take an anticoagulant (warfarin, dabigatran or rivaroxaban) for at least 3 weeks. A transesophageal echocardiography was performed on all patients to verify that no thrombus was present and a pulmonary vein CT to ensure that no significant variation was present among the patients. All 67 AF patients were subjected to RFCA by applying the ablation power at 35 watts with a temperature of 43 °C at the tip of the catheter. The left and right pulmonary vein ostia were ablated under the guidance of 3D electro-anatomical mapping systems (CARTO). The end point of RFCA was the elimination or isolation of pulmonary vein potential throughout the ostial circumference. If AF continued, a 120 J synchronized cardioversion was performed after a transvenous administration of 150 mg cordarone, to achieve sinus rhythm. Morphine was used to alleviate pain during the procedure. All the cases we selected were treated with the same drugs after RFCA.

### Follow-up examination

Transthoracic echocardiography was performed immediately after RFCA to exclude the existence of pericardial effusion. Follow-up of patients included detailed inquiries regarding arrhythmia-related symptoms (dizziness, chest distress or cardiopalmus) and echocardiography examinations. A 24-h Holter was performed at 3, 6 and 12 months after the ablation to confirm the presence or absence of AF recurrence. Based on these follow-up results, preoperative echocardiography parameters of the patients were retrospectively analyzed.

### Statistical analysis

Continuous data were expressed as Mean ± SD and compared with use of Student t-tests. Categorical data were expressed as percents and compared with use of the Pearson’s chi-squared test, continuity correction chi-squared test or Fisher’s exact test, as appropriate. Risk estimations were evaluated using univariate and multivariate logistic regression models with the presence of AF recurrence as the dependent variable. The Bland-Altman method was used to compare the reproducibility of the two physicians to measure independent risk factors. All data were required to achieve a *P* <  0.05 to be considered as statistically significant. The SPSS 19.0 statistical software package was used to analyze these data.

## Results

### Patient population

Basic characteristics of the study sample were summarized in Table 1. No statistically significant differences in demographics or clinical risk factors were present between the AF and control group. Echocardiographic variables between the AF and control group are presented in Table 2. The LAD and LA maximum volume index of AF patients was significantly increased (*p* <  0.001). TE-e’、 E/e’(S)、 E/e’(L)、the Tei index and E/Vp were all increased in AF patients as compared with that of the control group (*p* <  0.05). PV-DT and IVRT were shorter in AF patients than the control group (*p* < 0.05). Of the 67 patients enrolled, 57 who had E/e’(S) ratios and 49 who had E/e’(L) ratios during 8 to 15 were analyzed, in which period E/e’ is indeterminate for the evaluation of left ventricular diastolic pressures. No significant differences between the two groups were obtained for LVEF, LV end-diastolic and end-systolic diameter.

**Table 1:** Basic characteristics of the study population

	AF group (*n* = 67)	Control group (*n* = 47)	*p* Value
Mean age (years)	58.00 ± 10.28	57.66 ± 10.39	0.863
Male sex (%)	64.18%(43)	59.57%(28)	0.618
Height(m)	1.69 ± 0.08	1.67 ± 0.07	0.175
Weight (kg)	72.21 ± 11.67	68.28 ± 10.33	0.066
Body mass index (kg/m^2^)	25.07 ± 2.81	24.35 ± 3.33	0.213
Clinical risk factors			
Smoke	40.30%(27)	34.04%(16)	0.498
Hypertension	43.28%(29)	38.30%(18)	0.594
Diabetes mellitus	11.94%(8)	14.89%(7)	0.646
Chronic Coronary artery disease	46.27%(31)	40.43%(19)	0.536

Data are expressed as mean ± SD *AF* atrial fibrillation

**Table 2:** Echocardiographic variables between the AF group and the control group

	AF group (*n* = 67)	Control group (*n* = 47)	*p* Value
LA diameter (mm)	37.77 ± 6.28	29.34 ± 3.59	< 0.001
LA maximum volume index (mL/m^2^)	41.43 ± 5.73	27.83 ± 4.05	< 0.001
TE-e’(ms)	36.95 ± 7.05	22.17 ± 5.29	< 0.001
E/e’(S)	9.30 ± 2.80	7.55 ± 1.49	< 0.001
E/e’(L)	7.67 ± 1.37	5.88 ± 1.36	< 0.001
Tei index	0.50 ± 0.28	0.27 ± 0.06	< 0.001
E/Vp	1.24 ± 0.47	1.07 ± 0.34	0.029
PV-DT (ms)	153.1 ± 18.7	179.4 ± 15.6	< 0.001
IVRT (ms)	64.45 ± 9.84	70.62 ± 11.64	0.003
LV ejection fraction(%)	59.58 ± 12.25	61.97 ± 9.13	0.195
LV end-diastolic diameter (mm)	45.09 ± 3.84	44.30 ± 4.17	0.300
LV end-systolic diameter (mm)	26.56 ± 3.92	25.92 ± 3.60	0.376

Data are expressed as mean ± SD *LA* left atrial; *TE-e’* time interval between onset of E and of e’; *PV-DT* deceleration time of pulmonary venous diastolic velocity; *IVRT* isovolumic relaxation time; *LV* left ventricular

### Ablation outcome

All 67 patients with AF underwent successful pulmonary vein isolation or elimination by RFCA. In our study, 2 patients with AF recurrence underwent secondary radiofrequency ablation at their own discretion.

### Follow-up outcome

At the one-year follow-up examination after ablation, 21/67 (31.34%) AF patients relapsed. In the patients without AF recurrence, 7 of them had arrhythmia-related symptoms but no Holter confirmation of AF recurrence 1 year after ablation and their symptoms gradually improved. Characteristics of patients with or without AF recurrence after ablation are summarized in Table 3. Demographics, duration/type of AF, renal function and clinical risk factors were similar between the two groups. Echocardiography parameters of two groups are presented in Table 4. TE-e’ and the Tei index of patients with recurrence were significantly increased as compared with that of the group without recurrence(*p* <  0.001). PV-DT of the recurrence was shorter than the group without recurrence(*p* = 0.046). The LA maximum volume index of the recurrence was higher than the group without recurrence(*p* = 0.027). No significant differences were present for LV ejection fraction, LV end-diastolic diameter, LV end-systolic diameter, LAD, PkAcc of the E velocity, E, e’ (S), e’ (L), E/Vp, IVRT and NT-pro BNP between the two groups. Nor were the dual Doppler echocardiography parameters, E/e’(S) and E/e’(L) statistically significant between the groups with or without recurrence.

**Table 3:** Characteristics of patients with and without AF recurrence after ablation

	Recurrence		*p* Value
	Yes(*n* = 21)	No(*n* = 46)	
Mean age (years)	59.71 ± 10.42	57.22 ± 10.24	0.360
Male sex (%)	66.67%(14)	63.04%(29)	0.774
Height(m)	1.70 ± 0.08	1.69 ± 0.07	0.473
Weight (kg)	72.57 ± 11.25	72.04 ± 12.00	0.865
Body mass index (kg/m^2^)	24.90 ± 2.42	25.15 ± 3.00	0.735
Duration of AF (years)	3.93 ± 3.26	3.71 ± 3.91	0.827
Blood urea (mmol/L)	5.70 ± 1.30	5.80 ± 1.65	0.812
Creatinine (umol/L)	84.22 ± 16.84	88.03 ± 36.37	0.650
Type of AF			
Paroxysmal	76.19%(16)	82.61%(38)	
Persistent	14.29%(3)	13.04%(6)	0.770
Long persistent	9.52%(2)	4.35%(2)	
Clinical risk factors			
Smoke	52.38%(11)	34.78%(16)	0.173
Hypertension	47.62%(10)	41.30%(19)	0.628
Diabetes mellitus	4.76%(1)	15.22%(7)	0.413
Chronic Coronary artery disease	38.10%(8)	50.00%(23)	0.365

Data are expressed as mean ± SD

**Table 4:** Echocardiography parameters of patients with and without AF recurrence after ablation

	Recurrence		*p* Value
	Yes(*n* = 21)	No(*n* = 46)	
TE-e’(ms)	45.48 ± 6.17	33.06 ± 2.55	< 0.001
Tei index	0.76 ± 0.40	0.39 ± 0.03	< 0.001
PV-DT (ms)	146.4 ± 16.4	156.2 ± 19.1	0.046
LA maximum volume index (mL/m^2^)	44.19 ± 7.26	40.17 ± 4.42	0.027
LV ejection fraction(%)	58.54 ± 12.80	59.60 ± 12.13	0.985
LV end-diastolic diameter (mm)	45.01 ± 4.08	45.12 ± 3.77	0.911
LV end-systolic diameter (mm)	26.70 ± 3.81	26.49 ± 4.01	0.838
LA diameter (mm)	38.20 ± 7.07	37.58 ± 6.00	0.713
PkAcc of the E velocity (cm/sec2)	1943 ± 176	1942 ± 211	0.985
E (cm/s)	77.81 ± 19.00	72.45 ± 17.96	0.270
e’(S)(cm/s)	8.74 ± 2.51	8.42 ± 1.57	0.526
e’(L)(cm/s)	11.24 ± 2.70	9.96 ± 2.48	0.062
E/Vp	1.21 ± 0.48	1.26 ± 0.47	0.670
IVRT (ms)	66.71 ± 10.98	63.41 ± 9.21	0.205
NT-pro BNP (pg/mL)	297.9 ± 90.63	259.7 ± 61.61	0.090
E/e’(S)	9.69 ± 2.80	8.73 ± 0.79	0.137
E/e’(L)	7.56 ± 1.60	7.71 ± 1.27	0.680

Data are expressed as mean ± SD *PkAcc* peak acceleration

### Univariate and multivariate analysis of AF recurrence

Univariate analysis of AF recurrence at 1 year of follow-up was shown in Table 5. LA maximum volume index and TE-e’ were associated with AF recurrence at 1 year after radiofrequency ablation. Results from multivariate analysis for AF recurrence after ablation indicated that TE-e’ was significantly associated with AF recurrence as determined at 1 year after RFCA. Accordingly, TE-e’ can serve as an independent predictor for AF recurrence after RFCA (odds ratio: 2.59; 95% confidence interval:1.45–4.64; *p* = 0.001) (Table 5).

**Table 5:** Univariate and multivariate analysis for AF recurrence after ablation

	Univariate analysis		Multivariate analysis	
	OR(95% CI)	*p* Value	OR(95% CI)	*p* Value
Mean age (years)	1.03 (0.97–1.08)	0.356		
Male sex (%)	1.17 (0.40–3.48)	0.774		
Body mass index (kg/m^2^)	0.97 (0.80–1.17)	0.730		
Duration of AF (years)	1.02 (0.88–1.17)	0.824		
LA diameter (mm)	1.02 (0.94–1.10)	0.709		
LV ejection fraction (%)	1.00 (0.96–1.04)	0.985		
E (cm/s)	1.02 (1.00–1.05)	0.269		
e’(L)(cm/s)	1.23 (1.00–1.52)	0.067		
E/e’(L)	0.92 (0.63–1.35)	0.675		
e’(S)(cm/s)	1.09 (0.83–1.43)	0.521		
E/e’(S)	1.42 (0.99–2.06)	0.060		
TE-e’(ms)	2.59 (1.45–4.64)	0.001	2.59 (1.45–4.64)	0.001
IVRT (ms)	1.04 (0.98–1.10)	0.205		
NT-proBNP (pg/mL)	1.01 (1.00–1.02)	0.056		
PkAcc of the E velocity (cm/sec^2^)	1.00 (0.997–1.003)	0.984		
LA maximum volume index (mL/m^2^)	1.14 (1.03–1.27)	0.012		
PVDT (ms)	1.00 (0.939–1.000)	0.051		

Odds Ratio (95% CI) and relative computed *p* value were assessed *OR* odds ratio; *CI* confidence interval

### Reproducibility

**Fig. 6:**
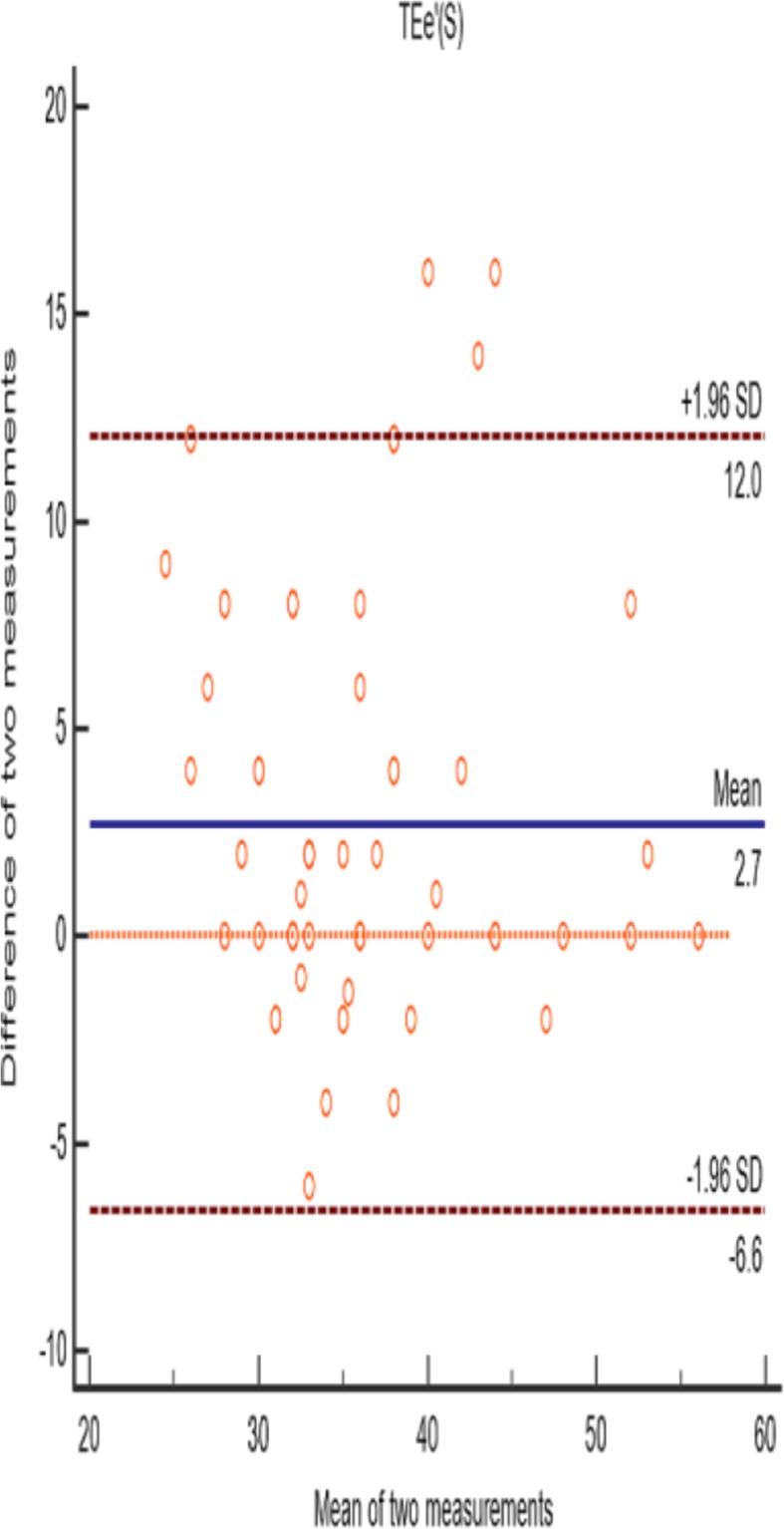
Bland Altman analysis of two physicians’ measure for TE-e’. 64 points were within 95% confidence interval, accounting for 95.52%

Bland Altman analysis of two physicians’ measure for TE-e’ was shown in Fig. 6. The intra-class correlation coefficient of TE-e’ was 0.84, 95% confidence interval is 0.65–0.92.

## Discussion

With use of dual Doppler echocardiography, we have demonstrated that TE-e’, E/e’(S), E/e’(L) and the Tei index in patients with AF were all significantly increased as compared with that observed in control patients with sinus rhythm. TE-e’ and the Tei index increased in patients with AF recurrence versus those without recurrence after ablation. TE-e’ obtained with use of dual Doppler echocardiography was found to be an independent predictor of AF recurrence at 1 year after RFCA. These findings suggest that TE-e’ could serve as an integrating marker for various risk factors of AF, and as an independent predictor for the recurrence of AF after RFCA. To the best of our knowledge, this study represents the first report in which TE-e’ has been demonstrated to function as a predictor of AF recurrence as determined at 1 year after RFCA.

LV diastolic dysfunction plays an important role in the development of AF [14, 15] and echocardiography is one of the most common methods used to evaluate LV diastolic dysfunction. IVRT and PV-DT have a good correlation with LV diastolic dysfunction [16, 17]. According to the American Society of Echocardiography and the European Association of Cardiovascular Imaging guidelines and standards, E/e’ ratio, TE-e’ and E/Vp can be used to identify patients with diastolic dysfunction [6]. While a Doppler index, combining systolic and diastolic time intervals (Tei index) has been reported to be useful for assessing global LV function and predicting clinical outcomes in adult patients with LV dysfunction [11], AF patients cannot use these indicators to accurately assess LV diastolic function due to the highly variable cycle lengths present in these patients. Dual Doppler echocardiography represents a relatively new technology which enables a simultaneous evaluation of two Doppler sample lines. In this way, a simultaneous observation of any changes in blood flow or myocardial tissue movement in the heart chamber can be assessed. Accordingly, this technique greatly improves the diagnostic efficiency for evaluating cardiac function in patients with arrhythmias [10].

Li et al. have demonstrated that E/e’ and LV filling pressure are highly correlated in patients with AF, particularly in the dual-Doppler mode [18]; and Kusunose et al. have reported that single-beat E/e’(L) was significantly correlated with pulmonary capillary wedge pressure [19]. Moreover, dual Doppler echocardiography has the capacity to simultaneously record E and e’ in AF patients and, with the combined analysis of TE-e’ and E/e’, an improved accuracy in LV filling pressure evaluation was achieved [10]. Accordingly, the feasibility of using E/e’ and TE–e’, as obtained with use of a dual Doppler system, was possible for use in the evaluation of LV filling pressure in patients with AF and a wide range of LVEFs. The results of Takasaki et al. suggest that the Tei index, combining systolic and diastolic function, and showing significant correlations both with pulmonary capillary wedge pressure and cardiac index, enabled a better evaluation of cardiac function [20]. Our current results demonstrating increased levels of E/e’(S), E/e’(L),the Tei index and TE-e’ within the AF group are in good agreement with these previous studies [10, 18–20].

LA maximum volume index is one of the important parameters for LV diastolic function rating [21]. In our study, LA maximum volume index of the AF group was higher than the control group, and the parameter increased in the AF recurrence group than the group without recurrence, which was consistent with previous studies [21, 22].

Since AF is a heterogeneous disease, the use of simple and objective parameters to identify high-risk groups for AF recurrence may be helpful in tailoring patient-specific therapeutic strategies. Factors such as interatrial conduction time, neutrophil/lymphocyte ratio and left atrial pressure have all been previously reported as predictors of AF recurrence after ablation [23–25]. However, with regard to echocardiographic predictors, no single parameter enables a prediction of AF relapse after catheter ablation [26].

In the present study, neither duration of AF, LAD, LV ejection fraction, E, e’, E/e’, IVRT, NT-proBNP, PkAcc of the E velocity or PV-DT predicted recurrence of AF. Our univariate analysis has showed that LA maximum volume index and TE-e’ were associated with AF recurrence 1 year after radiofrequency ablation, consistent with previous studies [24, 27]. Hirai has demonstrated that the E/e’ ratio, which is indicative of increased left atrial pressure, may serve as a marker for AF recurrence after ablation [4]. In our study, with use of dual Doppler echocardiography and multivariate analysis we found that TE-e’ provides an independent predictor of AF recurrence after ablation. It seems likely that the irregular heart rhythms of AF patients precludes conventional echocardiography from providing an accurate evaluation of their LV diastolic function.

The results of Sohn et al. have led to the suggestion that prolongation of TE-e’ might be involved with an elevated filling pressure in the setting of prolonged tau [28]. Moreover, it has also been reported that correlations obtained between TE-e’ and LV end-diastolic pressure in patients with normal systolic function and indeterminate E/e’ ratios appear to be stronger than those obtained from correlations between pro-BNP and LV end-diastolic pressure [29]. Our current results are in good agreement with these previous studies [10, 28, 29]. In addition, our study demonstrated that TE-e’ had good reproducibility. Specifically, here we demonstrate that TE-e’ of the AF recurrence group was significantly greater than that observed in the group without recurrence. In this way, TE-e’ can serve as an independent predictor for AF recurrence after ablation.

### Study limitations

Three notable limitations of our study should be acknowledged. The first being that this study involved a single-center, retrospective evaluation for AF recurrence. Second, the limited sample size of our study precluded our ability to achieve the necessary cutoff value of TE-e’ for prediction of recurrence. Third, the one-year follow-up of patients after ablation in this study may underestimate the recurrence rate of AF, because only intermittent 24-h Holter records and follow-up may lead to the omission of asymptomatic recurrence patients during clinical follow-up.

## Conclusions

Dual Doppler echocardiography can provide an effective and accurate technique for evaluating LV diastolic function within AF patients. The TE-e’ obtained within identical cardiac cycles can serve as an independent predictor for the recurrence of AF as determined at 1 year after ablation.

## Data Availability

The datasets used and/or analysed during the current study are available from the corresponding author on reasonable request.

## Abbreviations

AF: Atrial fibrillation
LAD: Left atrial diameter
LV: Left ventricular
LVEF: Left ventricular ejection fraction
PkAcc: Peak acceleration
PV-DT: Deceleration time of pulmonary venous diastolic velocity
RFCA: Radiofrequency catheter ablation
